# Advances in breath-hold diving research: a state-of-the-art review

**DOI:** 10.1007/s00421-025-06093-6

**Published:** 2025-12-19

**Authors:** Matteo Paganini, Richard E. Moon, Enrico M. Camporesi, Gerardo Bosco

**Affiliations:** 1 Department of Biomedical Sciences, University of Padova, Via Marzolo 3, 35131 Padova, PD Italy; 2 TEAMHealth Research Institute, Tampa General Hospital, Tampa, FL USA; 3 Center for Hyperbaric Medicine and Environmental Physiology, Department of Anesthesiology, Duke University Medical Center, Durham, NC USA

**Keywords:** Breath-hold diving, Environmental physiology, Apnea, Decompression stress, Cardiovascular adaptations, Underwater, Diving physiology

## Abstract

**Background:**

Breath-hold diving (BHD, also referred to as freediving) represents an extreme physiological challenge, requiring adaptations to rapid changes in blood gas levels and hydrostatic pressure. Despite advances in understanding human responses to BHD, knowledge gaps remain. With this state-of-the-art review, research trends and progression were tracked to inform future investigation directions.

**Methods:**

A structured literature search was conducted in PubMed and Scopus (2005–2025), selecting peer-reviewed studies on physiological, biochemical, and biomechanical aspects of BHD. Thematic analysis identified eight major research areas: cardiovascular, pulmonary, and neurological systems, decompression stress, skeletal muscle and metabolism, training, long-term adaptations, and technological advancements.

**Results:**

Cardiovascular adaptations involve autonomic regulation, bradycardia, and splenic contraction, but uncertainties remain regarding individual variability. Pulmonary responses include lung compression, gas exchange inefficiencies, and potential risks of lung barotrauma. Neurological effects include hypoxia-induced syncope, cerebral blood flow changes, and emerging evidence of neurovascular damage. Decompression stress, once considered negligible, is now recognized in elite and repetitive divers. Training enhances apnea performance through hematological and metabolic adaptations, though long-term effects are unclear. Telemonitoring advancements are promising for future improvement of divers’ safety.

**Conclusions:**

Recent observations emphasize both adaptive and maladaptive aspects of BHD physiology. The synthesized research trends should aim at refining current achievements and identify what individual and environmental factors pose specific limits for human breath-hold performance underwater.

**Supplementary Information:**

The online version contains supplementary material available at 10.1007/s00421-025-06093-6.

## Background

Breath-hold diving (also known as freediving) represents one of the most extreme physiological challenges the human body can endure. While rooted in ancient subsistence practices (Ilardo and Nielsen 2018), modern freediving has evolved into a high-performance sport and an experimental model for studying human adaptations to hypoxia and sudden interruption of breathing (Tetzlaff and Muth 2024).

Over the past two decades, our understanding of human physiology underwater has progressively improved (Bosco et al. 2007; Lindholm and Lundgren 2009; Bosco et al. 2018b; Fitz-Clarke 2018). Technological advancements have revolutionized the ability to explore the body’s adaptive responses to breath-hold diving, especially in critical aspects such as cardiovascular regulation, lung mechanics, and neurophysiological resilience (Dujic et al. 2011; Dujic and Breskovic 2012; Dujic et al. 2013; Patrician et al. 2021a). The interplay between evolutionary adaptations and plastic physiological responses raises fundamental questions about training (Ostrowski et al. 2012), long-term maladaptations (Elia et al. 2021), and the emerging risks associated with elite performance and competitive apnea (Muth and Tetzlaff 2024), to define the boundaries of safe performance and when adaptation may subtle shift towards pathology. Dives exceeding 200 meters and breath-hold duration surpassing 10 minutes were achieved after advances in training and using techniques such as hyperventilation or glossopharyngeal insufflation (GI)—introducing significant physiological trade-offs potentially leading to life-threatening acute pathologies or long-term health consequences affecting the neurological, cardiovascular, and pulmonary domains (Mijacika and Dujic 2016; Tetzlaff et al. 2021). A better understanding of decompression stress also opened new interpretations of decompression illness and depth narcosis in breath-hold diving (Schipke et al. 2006; Patrician et al. 2021b; Blogg et al. 2023). The body-mind interconnection has been further explored, conceptualizing freediving as an embodied skill-building process where physiological adaptation, cognitive learning, and sensory attunement converge through deliberate practice to refine apnea-related motor control and autonomic regulation (Downey 2024).

This state-of-the-art review comprehensively analyzes breath-hold diving literature to synthesize the most recent achievements, delineating how we obtained such knowledge in the last 20 years and where research is pointing to fill the identified gaps.

## Methods

This state-of-the-art review uses a structured methodology to identify and synthesize the advancements in breath-hold diving research over the past twenty years. The approach integrates a conceptual and historical progression model, emphasizing shifts in scientific understanding, key turning points, and persisting research gaps (Barry et al. 2022a).

### Literature search strategy

We conducted a targeted search across PubMed and Scopus of peer-reviewed literature published in the last 20 years. A combination of controlled vocabulary (e.g., MeSH terms) and free-text keywords was employed, focusing on breath-hold diving, human physiology, underwater environments, and adaptive mechanisms (Grant and Booth 2009). Four weeks before submission, another search was performed to capture potential other recent publications.

Unlike systematic reviews, which aim for exhaustive coverage, this approach selected key contributions that reflect the evolution of scientific thinking rather than a rigidly structured dataset (Barry et al. 2022a, b). The search prioritized seminal studies, high-impact publications, and influential reviews that have shaped the contemporary understanding of breath-hold diving physiology. Case reports and case series were included if they provided evidence that was lacking in specific fields. Studies were finally selected after consensus among the authors, if published in peer-reviewed journals, focusing on physiological, biochemical, or biomechanical aspects of breath-hold diving and having investigated humans. Studies not available in English, non-peer-reviewed literature, and research focused on techniques other than breath-hold diving were excluded.

### Approach: data extraction, thematic analysis and synthesis

Key data extracted from eligible studies included—where available—study design, sample characteristics, experimental protocols, environmental conditions, physiological variables measured, and main findings.

Thematic analysis was then employed to synthesize research findings, allowing for the identification of emerging trends and research gaps. The analysis followed an iterative process as suggested by Naeem et al (Naeem et al. 2023). Initially, full-text reviews were conducted to extract key physiological and methodological insights. Through repeated readings, patterns inductively emerged, forming a foundation for thematic coding. These extracted themes were refined and categorized into major physiological and environmental domains by consensus among the authors, ensuring coherence with seminal studies and recent advancements. In order to finally describe and discuss the results, the three-part framework of a state-of-the-art review was adopted: (1) This is where we are now, (2) This is how we got here, and (3) This is where we could go next (Barry et al. 2022c).

Data were coded onto a master sheet using a Microsoft Office Excel spreadsheet (Version 2016, Microsoft Corporation, Redmond, WA).

## Results

**Fig. 1:**
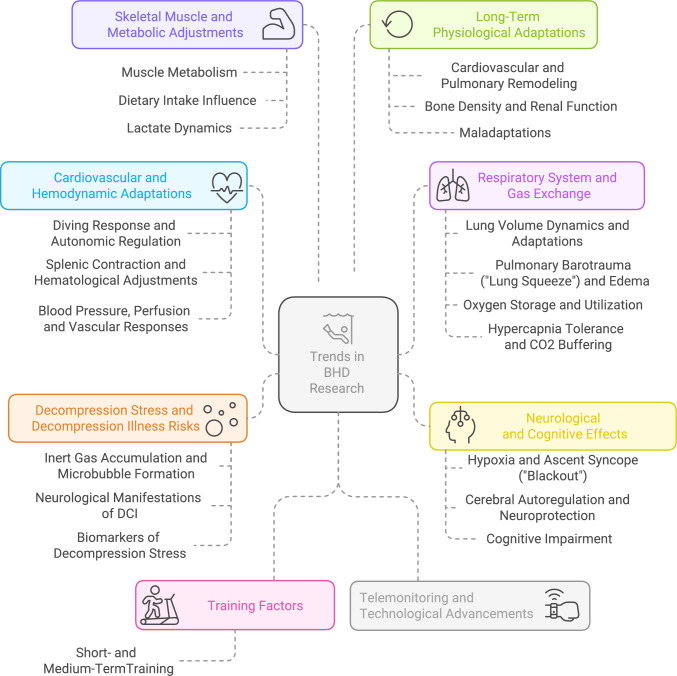
Eight major themes and pertinent subthemes identified among the breath-hold diving physiology papers included

A structured literature search was conducted in PubMed and Scopus from January 6 to February 11, 2025, using the combination of keywords available as Supplementary Material 1. A total of 381 records were retrieved. After removing duplicates, 187 unique articles remained for the title and abstract screening, leading to 161 studies selected. Further 5 studies were added after a final search, totaling 166 studies included. After reading the papers, a guide was structured to ensure thematic analysis consistency and identify themes and subthemes emerging from the literature (Supplementary Table 2). Eight final themes were identified: the cardiovascular, pulmonary, and nervous systems, decompression stress and decompression illness (DCI), skeletal muscle and metabolism, training factors, long-term adaptations, and telemonitoring and technological advancements (Fig. 1; Supplementary Material 3). These themes were then mapped against historical research developments to track the evolution of scientific understanding in the field and identify gaps.

## Discussion

### Cardiovascular and hemodynamic adaptations

This theme included the highest number of selected papers (46), confirming its complexity, with 42 original research papers, two case reports, and two reviews by Malinowksi et al. (Malinowski et al. 2025) and by Arce-Álvarez et al (Arce-Álvarez et al. 2022).

Autonomic regulation during breath-hold diving is characterized by an orchestrated balance of sympathetic activation and parasympathetic dominance, manifesting as bradycardia, peripheral vasoconstriction, increased mean arterial blood pressure (MAP), splenic contraction, and higher hemoglobin concentration. In simulated (dry) static apnea, 20 elite divers vs. 15 untrained participants showed a biphasic sympathetic activation: an early-phase triggered by baroreflex and increased intrathoracic pressure; and a late-phase excitation driven by chemoreflex (linked to hypoxia, hypercapnia, and lack of sympathetic ventilatory inhibition) (Heusser et al. 2009). In a similar setting, the introduction of glossopharyngeal insufflation (GI) further augmented sympathetic activation and dampened early arterial pressure increase, likely due to hypothesized overinflated lung-released mediators or measurement artifacts (Heusser et al. 2010). However, both studies lack underwater exposure, limiting their conclusions.

Heart rate variability (HRV) is an indicator of autonomic regulation, reflecting the balance between sympathetic and parasympathetic activity. Kiviniemi et al. found that, despite higher heart rates and sympathetic activation during dynamic apnea (surface), HRV at end-apnea was comparable to static underwater breath-hold, suggesting vagal modulation against exercise-induced sympathetic activation in nine elite divers (Kiviniemi et al. 2012). Ten years later, Vitali et al. compared dry vs. wet warm-up protocols, finding that dry warm-up induces greater autonomic activity during dynamic apnea (Vitali et al. 2022). In general, bradycardia is induced by static apnea submerged in cold water (Perini et al. 2010), by isolated face immersion in cold water (Baković et al. 2005; Solich-Talanda et al. 2019), or even by dry apnea alone (Solich-Talanda et al. 2019; Marlinge et al. 2021; Persson et al. 2023). Submersion without breath-holding, instead, did not induce bradycardia, independently from water temperature variation on the face (Wester et al. 2009). The magnitude and timing of heart rate reduction seem workload-dependent, with higher workloads delaying and attenuating the predicted bradycardia. For example, in experienced breath-hold divers, heart rate was higher during dynamic apnea than static apnea (wet) (Breskovic et al. 2011), exercising with face immersion (Wein et al. 2007), or secondary to exercise during open-sea dives (Marongiu et al. 2015). Among nine Haenyeo divers, heart rate decreased compared with surface swimming but did not reach bradycardia—findings limited by the small sample size and the divers’ life-long adaptations (Lee et al. 2016). Under dry simulated conditions, cycling during dynamic apneas partially blunted the early bradycardia in recreational divers, but by end-apnea heart rate similarly decreased (Allinger et al. 2025). Brown et al. registered lower peak heart rates during maximal dry dynamic apneas on a swim-ergometer than static apneas, but in moderate and elite freedivers (Brown et al. 2024). Bradycardia did not develop in the first but only in a second and a third dive with fins to 11 mfw, potentially related to repeated dive adaptations (Mulder et al. 2023b). Peak heart rate was also higher if face-immersed apnea was preceded by hyperventilation than without in non-divers, potentially indicating parasympathetic suppression and increased metabolic demand during preparation (recruitment of accessory respiratory muscles) (Pernett et al. 2023); this result can however vary in divers, due to training effects. GI caused transient sinus tachycardia before the dive but did not blunt the diving-induced bradycardia, as measured with a marinized ECG on two deep sea dives (Patrician et al. 2021b) or indirectly through near-infrared spectroscopy (NIRS) placed on the head in open-sea dives (McKnight et al. 2021).

Splenic contraction has traditionally been ascribed primarily to hypoxemia and hypercapnia-induced chemoreceptor activation, triggering the sympathetic response (Arce-Álvarez et al. 2022). This was probably sustained by previous evidence showing that dry apnea induces increased hematocrit and hemoglobin concentration due to splenic contraction but is not influenced by facial immersion (Schagatay et al. 2007). Persson et al. confirmed the role of the sympathetic system during dry apnea, suggesting that respiratory arrest is a potent initiator of splenic contraction in a crossover comparing 5 periods of dry apnea vs. rebreathing (Persson et al. 2023). The work of Badrov et al. is consistent with Persson et al. since, in their protocol, restored ventilation suppresses sympathetic stimuli despite high chemoreflex stress (Badrov et al. 2017). Non-trained participants as well demonstrate splenic contraction, which is not influenced by hyperventilation preceding face-immersed apnea (Pernett et al. 2023).

Arterial blood pressure is one of the most studied outcomes within the included papers, which overall tend to confirm its rise. With a mathematical model including lung packing and the blood shift among compartments as a variable, Fitz-Clarke describes an increase in blood pressure while descending, after an initial blunted rise due to GI (Fitz-Clarke 2007b). As a primary outcome, Sieber et al. measured arterial blood pressure (systolic and diastolic) in static apnea at 10 mfw (26°C), finding no significant increase (Sieber et al. 2009). Other groups found instead a considerable rise in static dry (Marlinge et al. 2021), static (head submersed) apnea (Perini et al. 2010; Breskovic et al. 2011), and dynamic apnea to 2 mfw (Breskovic et al. 2011). All of these studies conflict with extremely elevated pressures (280/200 and 290/150 mmHg) previously found on two elite divers by Ferrigno et al. in 1997 in a simulated dive to 50 m in a hyperbaric chamber (water: 25 °C), which was criticized and subsequently commented by the authors (Ferrigno and Lundgren 2010). As a secondary outcome, compared to non-divers, elite divers showed a rise in arterial pressure (Wein et al. 2007; Persson et al. 2023; Brown et al. 2024) and higher peripheral vascular resistance (Heusser et al. 2009), but in simulated (dry static or dynamic) conditions. Fico et al. reproduced simulated breath-hold dives, confirming a rise in arterial blood pressure and peripheral vascular tone in non-divers (Fico et al. 2022). Higher increase in blood adenosine levels were measured in spearfishermen and not in controls after static dry apnea, along with a more pronounced bradycardia and higher systolic blood pressure values—suggesting a role of this metabolite in modulating apnea-related hypertension to maintain blood flow to vital organs (Marlinge et al. 2021). The introduction of GI before static (dry) apnea paradoxically dampened early arterial pressure increase and peripheral vascular resistance, likely due to reduced venous return and cardiac output from excessively elevated intrathoracic pressure (Heusser et al. 2010). This blood pressure drop at the beginning of dry apnea when using GI was further confirmed (Schiffer and Lindholm 2013; Boussuges et al. 2014) leading to syncope in two experimental participants (Schiffer and Lindholm 2013), as predicted by Fitz-Clarke (Fitz-Clarke 2007b).

Cardiac output is reduced in divers during dry apnea (Persson et al. 2023) and during maximal dry dynamic apneas on a swim-ergometer (more in elite than in recreational divers) (Brown et al. 2024). A slightly significant rise was instead found by Fico et al. during apneic, face-immersed exercise on a cycle ergometer performed by non-divers, but blunted if compared to exercise alone, exercise with facial immersion (snorkel breathing), and apneic dry exercise (Fico et al. 2022). Echocardiographic underwater studies in male experienced divers at 5 and 10 mfw without GI revealed cardiac output reduction and a restrictive left ventricular diastolic dysfunction, probably induced by reduced chest volume plus chest blood pooling (Marabotti et al. 2009a, b). This hypothesis was confirmed by breathing compressed air at 10 mfw from a SCUBA circuit that partially reversed systolic and diastolic dysfunction (Marabotti et al. 2009b). Similarly, cardiac output was reduced during the static phase at the bottom of an open-sea dive and increased during active descent and ascent with fins (Marongiu et al. 2015). Supporting the role of autonomic modulation, the administration of esmolol—a selective, short acting β-1 blocker—to elite divers isolated the contribution of β-adrenergic drive to cardiac performance, reducing stroke volume and consequently cardiac output during dry static apnea, while arterial blood pressure was maintained (probably due to increased sympathetic alpha receptor compensation) (Hoiland et al. 2017). The introduction of GI led to different results. Heusser et al. did not find any difference in cardiac output reduction during dry static apnea with and without GI in trained divers (Heusser et al. 2010). Instead, Fitz-Clarke estimated cardiac output reduction in his breath-hold diving model (Fitz-Clarke 2007b), an assumption confirmed by Boussuges et al. in dry conditions compared to end-inspiratory apnea probably due to the elevated intra-thoracic pressure developed by the two included elite breath-hold divers performing the maneuver (Boussuges et al. 2014). Consistently, Seccombe et al. identified reduced lung perfusion after GI with SPECT scans (Seccombe et al. 2010). Using cardiac magnetic resonance imaging (MRI) Schipke et al. demonstrated decreased venous return and stroke volume after GI in an elite female breath-hold diver (Schipke et al. 2015). In 2017, Mijacika et al. explored with thoracic MRI and ultrasound after GI the consistent drop in pulmonary and central blood volume after a decrease in cardiac chamber volumes and blood pooling in the extrathoracic capacitance veins—proximal to venous valves (Mijacika et al. 2017a, b). McKnight et al. indirectly explained drops in cerebral tissue saturation index registered with NIRS after GI as related to a reduction in cardiac output (McKnight et al. 2021). In 2024, Kjeld et al. confirmed these results with a methodology integrating echocardiography, cardiac MRI, and PET/CT scans (Kjeld et al. 2024). Interestingly, 1 h after completing a spearfishing competition, severe dehydration caused a reduction of arterial pressure and cardiac output while increasing HRV, denoting sympathetic activation (Gargne et al. 2012).

An increasing trend was noted in papers trying to address sex differences in cardiovascular responses. Splenic contraction showed sex-based variability in non-divers—with men exhibiting larger spleen size and more significant absolute splenic contraction than women (Pernett et al. 2024b). Despite this, hematological variations triggered by dry dynamic apnea seems similar between sexes of breath-hold divers (Brown et al. 2024). According to Malinowski et al.—gathering literature investigating cardiovascular responses during facial cooling—men seem to have more pronounced increases in arterial blood pressure and peripheral resistance (Malinowski et al. 2025). Cherouveim et al. compared non-divers during a maximal effort with face-immerged apnea, finding higher peripheral vascular resistance and lower cardiac output in males (Cherouveim et al. 2013). In contrast, Magnani et al. found a more significant cardiac output decrease in females, explained by a reduction in stroke volume after a steeper increase in systemic vascular resistance; the difference could derive from the different samples (here elite breath-hold divers, matched for experience in breath-hold dives) and protocols (here dry apneas, no face immersion) (Magnani et al. 2018). Cherouveim et al. also found no effect of sex on heart rates and blood pressure variations (Cherouveim et al. 2013). Sex differences in HRV account for higher short-term HRV indices in women (Malinowski et al. 2025), without differences in percentage heart rate reduction between the two sexes during dry apnea in 81 non-divers (Pernett et al. 2024b).

Despite such extensive research, the cardiovascular system has been an intriguing interest. Future research should refine the understanding of the individual variability in diving reflex and autonomic regulation, especially regarding sex differences and training- or workload-related modifications. Also genetic predisposition is still unexplored, with only a comparative genomic study available demonstrating significantly larger spleens of Bajau fishermen compared to neighboring populations due to positive selection on the PDE10A gene (Ilardo et al. 2018). Attention should be paid to elite performers, reaching depths below 100 meters or prolonged static breath-hold times, and to techniques like GI/hyperventilation, to ultimately improve safety. Thanks to technological advances, a visible trend towards underwater, real-time studies and monitoring in the last decade promises to produce literature with better external validity and fewer inconsistencies arising from simulated dry conditions, which are now unfortunately prevalent due to feasibility issues.

### Respiratory system and gas exchange

Fourty papers were included in this theme: 34 original research papers, 5 case reports, and one review by Foster and Sheel (Foster and Sheel 2005). This review confirms that, as with cardiovascular adaptations, the ventilatory response in breath-hold divers has intrigued researchers for a long time.

Regarding baseline parameters, elite divers tend to have higher total lung capacity (TLC), predicted forced expiratory volume in 1 second (FEV1), forced vital capacity (FVC), vital capacity (VC), and alveolar volume (Seccombe et al. 2010; Kjeld et al. 2024). Compared to controls, the absolute diffusion capacity for carbon monoxide was similar, but divers exhibited a lower diffusion constant when adjusted for the higher alveolar volume (Kjeld et al. 2024). While these findings highlight the structural and functional adaptations of the respiratory system—further discussed in “*Long Term Physiological Adaptations*”—the search for deeper dives posed new compelling questions on how lungs react to extreme depths and repetitive diving, specifically dealing with lung barotrauma.

Fitz-Clarke’s computational models (2007 and 2009) provided foundational insights into pulmonary mechanics and gas exchange during breath-hold diving, predicting airway and alveolar collapse at extreme depths, identifying a critical depth (~300 m) beyond which pulmonary gas exchange becomes ineffective (Fitz-Clarke 2007a, 2009).

In addition to underwater lung compression caused by ambient pressure changes, current understanding of lung barotrauma pathogenesis implicates the cardiovascular responses elicited during breath-hold diving, leading to lung edema and hemoptysis. Specifically, after blood shift and pulmonary circulation engorgement, an increase in pulmonary vascular pressure is expected, along with alveolar pressures lower than the environmental, developing when the lungs are compressed to residual volume; the consequent extravasation of fluids causes lung edema and smaller airway swelling. Hemoptysis derives from pulmonary capillary rupture secondary to capillary pressure-alveolar pressure difference exceeding capillary rupture thresholds, confirmed as originating from alveolar hemorrhage with bronchoscopy (Barković et al. 2021). Silva et al. also found petechiae on bronchoscopy, potentially supporting the hypothesis that diaphragmatic involuntary contractions during breath-hold could worsen the relative negative pressure of airways at depth (Silva et al. 2024). Pre-existing lung alterations can be responsible for hemoptysis even in shallow water breath-hold diving (3.6 mfw) (Inman et al. 2022). These three case reports, however, only suggest potential theories in hemoptysis pathogenesis, calling for further evidence. Lindholm et al. reproduced a model of thoracic squeeze by asking trained divers to perform glossopharyngeal exsufflation before repeated shallow depth dives (thus going below residual volume), and found obstructive alterations such as reduction of FVC and FEV1 but no significant changes in static spirometry indices; of note, two participants demonstrated fresh blood originating from below the vocal cords on laryngoscopy (Lindholm et al. 2008). Most of the elite divers included by Linér and Andersson demonstrated signs or symptoms of lung edema after diving to depths between 41 and 75 m, mirroring the reduction of FVC and FEV1, accompanied by a decrease in arterial oxygen saturation (Linér and Andersson 2008). In contrast, Seccombe et al. did not report significant changes in spirometry before and after a spearfishing competition, likely due to the shallower depths involved (average 10 msw; no water temperature available) (Seccombe et al. 2012), suggesting that depth remains a critical determinant in the development of diving-induced pulmonary alterations. More recently, elite breath-hold divers showed temporary respiratory symptoms and impaired gas exchange efficiency after two deep dives (>100 msw); also transient reduction in overall airway resistance and increase in smaller airway reactance after the dives sustained an increase in lung compliance (Patrician et al. 2021b), which potentially imply overdistension or atelectasis, and were consistent with extreme lung compression (calculated as above 90%). A correlation between pulmonary gas exchange inefficiency and depth—especially in those with estimated lung compression below residual volume—and with symptoms of pulmonary barotrauma was derived by Patrician et al. (Patrician et al. 2021d).

Lung edema or hemoptysis in the breath-hold diver can vary from overt, catastrophic manifestations to subtle symptoms; in the latter case, lung ultrasound proved to be a sensitive technique to monitor divers or perform differential diagnosis on site. Frassi et al. observed an increased number of B-lines (at that time called “lung comets”) in the lungs of elite divers performing deep dives (range 31–122 msw) (Frassi et al. 2008), consistent with pulmonary edema. Boussuges et al. identified a low incidence of B-lines in competitive fishermen after repeated dives (range 12–45 msw) in cold water; in those presenting B-lines (3 out of 30), respiratory symptoms were accompanied by spirometry alterations (Boussuges et al. 2011). Lambrechts et al. showed a significant increase in B-lines after both a dynamic surface and a deep (50 ± 10 msw) breath-hold dive in 42 experienced divers, concluding that the influence of environmental pressure is not mandatory to generate lung edema, rather than dive-related cardiovascular variations and diaphragmatic involuntary contractions (Lambrechts et al. 2011). In all these studies, lung scans were performed at about or within 10 minutes from the end of the dives. The number of B-lines was also positively correlated with the depth reached, with lower oxygen saturations and with more symptoms noted by Patrician et al. despite ultrasound scans performed 44 ± 15 min after the dives which limits the findings (Patrician et al. 2021c). In another study, B-lines were still doubled about 2.5 h after the first group of deep dives and were more prevalent in the right lung. In contrast, B-lines were more prevalent in a second group of deep dives but acquired about 9 minutes after resurfacing (Patrician et al. 2021d). More recent studies detected a higher incidence of B-lines performing lung scans immediately after the end of dives (Paganini et al. 2023; Yu et al. 2024), suggesting that earlier studies may have underestimated lung involvement due to delayed imaging and partial resorption of extravascular lung water. More severe presentations, including hemoptysis, were visualized as diffused interstitial edema and, interestingly, an area of atelectasis in the most affected apical lobe (Barković et al. 2021); this may suggest that lungs are asymmetrically subjected to environmental pressure in head-down descent and head-up ascent, with apical lobes being the first to be compressed and decompressed, respectively, but is limited to a single case. What has been more trending recently, in sight of potential long-term damage, is the study of lung hyperinflation after GI. Dynamic magnetic resonance imaging on an elite diver showed enlargement of the costodiaphragmatic angle and subxiphoid lung herniation after GI, increasing intrathoracic gas volume by 2.6 L (Eichinger et al. 2008). Similarly, thoracic CT scan in four elite divers showed that thoracic expansion (increased VC and TLC) after GI was related to caudal displacement of the diaphragm, intercostal bulging of lung tissue, and displacement of the vascular mediastinum; through SPECT scan, about 31% of the increase in expired lung volume was ascribed to gas compression (Seccombe et al. 2010). Added to environmental variables, divers can add a consistent strain on the respiratory system by using GI, which increases VC, TLC, and even residual volume, causing an impressive 97.3% rise in pulmonary airway pressures measured at surface while sitting (mean 7.26 ± 2.04 kPa; max value in one participant: 10.32 kPa) (Schiffer and Lindholm 2013).

Regarding gas exchange, early work by Baković et al. measured blood O_2_ and CO_2_ variations in 18 trained divers through transcutaneous measurement during repeated face-immersed breath-holds, confirming the predicted O_2_ reduction and CO_2_ accumulation, acknowledging the technique’s limitations (Baković et al. 2005). The gold standard to explore O₂ and CO₂ variations is, in fact, arterial blood gas (ABG) analysis, increasingly used in recent years despite its invasive nature and the technical challenges—though progressively mitigated—of obtaining underwater samples and ensuring timely processing (Paganini et al. 2022). Bosco et al. pioneered the first ABG analyses underwater, confirming hyperoxemia developing in only 4 out of 6 elite divers at − 40 mfw in warm pool water (Bosco et al. 2018a). An elite diver showed bottom arterial partial pressures of oxygen (PaO₂) reduction between the first and the second dive to 60 msw (Scott et al. 2021). Three participants also demonstrated variable results after a second dive to 10 msw after complete exhalation (Barković et al. 2023). Further work in warm water showed that several divers did not develop the predicted hyperoxemia at depth, potentially caused by lung atelectasis, supported by lung ultrasound performed within 1 minute after resurfacing (Paganini et al. 2023). The differences between pool and open-sea experiments—primarily colder water and more stressful sea conditions—may have elicited a stronger diving response in seawater, which could explain fewer cases of relative bottom hypoxemia observed at sea despite the smaller sample size. A reduction in PaO₂ was noted in several works when resurfacing (Bosco et al. 2018a, 2020; Scott et al. 2021; Paganini et al. 2023)—proportionally with the depth reached and physical effort, to values as low as 18 mmHg after using fins (Bosco et al. 2020). In the study reported by Barković et al., only one participant demonstrated resurfacing hypoxemia after a 60 mfw dive—but samples were drawn after resumption of breathing (Barković et al. 2023). Surface hypoxemia has also been recently shown after static apnea despite GI (Kjeld et al. 2024). Pulse oximeters non-invasively added insight to the above results, initially in simulated settings (Wein et al. 2007; Solich-Talanda et al. 2019). De Asís Fernández et al. divided 22 experienced male breath-hold divers into slow-recovery and fast-recovery groups according to the time needed to recover from surface hypoxia after a 30 msw dive. They found that the “hook breathing” method—deep inspiration without GI, followed by a Valsalva-like maneuver during expiration which is concluded exhaling against resistance—improved peripheral oxygen saturation faster than normal breathing in the slow-recovery group (de Asís Fernández et al. 2019b). Pulse oximetry values showed progressively worsening resurfacing values across 3 consecutive dives to 11 mfw without hyperventilation or GI (Mulder et al. 2023b), potentially due to over-the-time increased oxygen extraction in working muscles and no sufficient time to restore oxygen, or to accumulating stress on the lungs (again, in terms of lung edema or lung atelectasis). A similar trend is described by Pernett et al. after 5 hyperventilation and face-immerged apnea attempts (Pernett et al. 2023). Lastly, Persson et al. added to the long line of end-tidal gas measurement research showing that dry, supine apnea produces less end-tidal O_2_ depletion and a smaller rise in CO₂ than bag rebreathing, probably because it elicits a more pronounced cardiovascular response and associated oxygen-sparing mechanisms; this recent insight is however limited by the simulated setting (Persson et al. 2023).

Arterial partial pressures of CO_2_ (PaCO_2_) before diving highly depend on the type of preparation. During surface (immersed, head underwater) breath-hold, PaCO₂ showed a constant increase reflecting metabolic accumulation alone (Bosco et al. 2020; Scott et al. 2021; Kjeld et al. 2024). Hypocapnia was induced by GI (Scott et al. 2021) or by controlled, in-water ventilation without hyperventilation (Paganini et al. 2023). Bottom samples showed slight increases due to metabolic accumulation, returning to normal values—but slightly higher than the pre-dive—probably after lung re-expansion or tissue distribution (Bosco et al. 2018a; Scott et al. 2021; Barković et al. 2023; Paganini et al. 2023). Since CO₂ accumulation is a major limiting factor in breath-hold duration and triggers the urge to breathe, simulated (dry) investigations have sought to determine whether elite divers exhibit enhanced CO₂ tolerance compared to non-divers. By increasing inspired CO_2_ partial pressures, Binks et al. found that elite divers do not differ from non-divers regarding the urge to breathe and the hypercapnic ventilatory response (Binks et al. 2007). With a protocol of five face-immersed apneas, Andersson et al. found an increase in breath-hold duration without modifications in CO_2_ sensitivity as measured through hypercapnic ventilatory response (Andersson and Schagatay 2009). In a similar experiment, hyperventilation reduced post-apnea end-tidal CO_2_ and end-tidal O_2_, increasing breath-hold times against normal breathing and across five attempts (Pernett et al. 2023). Moving to a pool setting, end-tidal CO_2_ in underwater rugby players built up to values otherwise considered to cause impaired cognitive performance, while end-tidal O_2_ showed slight variations, supporting the fact that the athletes’ urge to breathe was regulated more by hypoxia than hypercapnia (Lindholm et al. 2022). Interestingly, by introducing a carbohydrate-free diet and shifting metabolism toward fat oxidation, prolonged dry exercise while breath-holding produced lower than expected end-tidal CO_2_ and O_2_ in eight experienced divers; such relatively reduced CO₂ production delayed the urge to breathe, exacerbating hypoxia and increasing the risk of loss of consciousness (Lindholm and Gennser 2005). On simulated dry apneas without and with effort including 50 healthy participants, the rise in end-tidal CO_2_ was confirmed as dependent on each athlete’s metabolic rate, hampering the imposition of a safety time limit for non-experienced breath-hold divers (Sadler et al. 2020).

Sex differences have also been explored limitedly in the respiratory system during breath-hold diving. Despite higher aerobic capacity and lung volumes in males, maximal-effort apnea durations were similar between sexes in non-divers, with or without face immersion in water (Cherouveim et al. 2013). In a sub-analysis of their 2021 study, Patrician et al. showed that males have a more significant after-dive gas exchange impairment (greater O_2_ deficit) than females; still, the low female sample size and deeper dives performed by males limit this result (Patrician et al. 2021d). More recently, Pernett et al. studied non-divers during two dry apneas, finding that, in men, VC is higher and—differently from Cherouveim et al.—positively correlated with apnea duration without any differences in peripheral oxygen saturation decrease (Pernett et al. 2024b). As well in experienced divers, % FVC (FVC corrected per height and age) positively correlated with maximal static apnea duration, and identifying men as better performers due to the higher % FVC; however, durations were not actually measured but self-declared by each participant, thus limiting the validity of this analysis (Peng et al. 2021).

Future research should deepen our understanding of lung barotrauma pathophysiology, specifically why lung edema and hemoptysis manifest with different degrees among breath-hold divers, in parallel with gas exchange study after repetitive dives, not only to high depths, since shallow water can also cause pulmonary capillary stress. The different clinical pictures including gas exchange modifications, resurfacing hypoxia, hypercapnia, lung edema, hemoptysis, atelectasis, and airway modifications demand more complex studies integrating multimodal research techniques. Lung ultrasound is a field-ready technique that can support both research and clinical practice by detecting subclinical pathology early and facilitating monitoring, particularly during diving competitions. However, in diving medicine and physiology it remains at an experimental stage, with the few available studies using heterogeneous techniques and timings (Nowak et al. 2025). As done for the diagnosis swimming-induced pulmonary edema (Hårdstedt et al. 2020, 2021), prospective studies including large numbers of breath-hold divers could help standardize and validate lung ultrasound protocols. Also, specific factors such as the environment (e.g., water temperature), phenotypic inter-individual variability, and different training and preparation for the dive should be investigated to explain the current variability in the results.

### Neurological and cognitive effects

Fourteen papers were included in this section—11 original research papers, one case report, one review, and one systematic review by Janigro et al. (Janigro et al. 2021)—linking acute variations in cerebral perfusion, syncope (“blackout”), transient loss of motor control (LMC), and markers of neuronal damage.

Historically, hypoxia-induced syncope—known as shallow water blackout—and LMC have been attributed to severe hypoxemia developing during the ascent phase due to rapid decreases in alveolar oxygen partial pressure. The relatively high prevalence of LMC and blackout in competitive settings has been linked to severe hypoxemia and hypocapnia induced by hyperventilation before apnea and the pursuit of extreme performances (Lindholm 2007; Lindholm and Lundgren 2009). The hypoxemia of the ascent was confirmed by several research groups, both invasively (Bosco et al. 2018a, 2020; Scott et al. 2021; Barković et al. 2023; Paganini et al. 2023) and non-invasively (Mulder et al. 2023c) as already described in the section *Respiratory System and Gas Exchange*. From a molecular point of view, Joulia et al. compared adenosine plasma concentrations (APC) in competitive breath-hold divers who had experienced at least 1 episode of syncope before vs. those who had not vs. controls, finding that the first group had higher APC at both basal levels and after a dry, static apnea. Since adenosine release is triggered by hypoxia, causing bradycardia and/or vasodilation, its link with ascent syncope can raise the suspicion of individual predispositions, despite the small sample (Joulia et al. 2013).

Another hypothesis involving cardiac arrhythmias and autonomic conflict behind blackouts is gaining importance. Mulder et al. reported alternation of bradycardia and tachycardia before syncope in a monitored athlete, leading to syncope probably after systemic and cerebral hypoperfusion (Mulder et al. 2023a). A similar mechanism has been suggested as the cause of drowning after immersion in cold water and cold-shock response (Shattock and Tipton 2012). Additionally, Poiret et al. demonstrated that slower speeds during the active descent phase correlated with syncope in constant weight without fins dives, probably due to poorer propulsive efficiency leading to higher oxygen consumption; faster speeds during the active descent phase in constant weight dives (with fins) also correlated with syncope risk, as allowed to achieve deeper and longer dives with potentially more severe hypoxia. These findings, obtained retrospectively reviewing videos collected during real international competitions, underscore the importance of optimizing descent technique efficiency as a preventive strategy against hypoxic blackout and LMC (Poiret et al. 2024).

From central nervous system’s perspective, oxygenation studies in elite divers using NIRS have shown significant cerebral tissue saturation index (TSI) decrease during the most prolonged apnea, paired with arterial oxygen saturation levels dropping to as low as 25% in open-sea deep dives (McKnight et al. 2021). Bønnelycke et al. in standardized pool dives to 15 and 42 mfw found less profound but similarly declining TSI during ascent, paired with slight reduction in cerebral blood volume and peripheral O_2_ desaturation (Bønnelycke et al. 2024). Finally, Allinger et al. found a greater imbalance between oxygen supply and pre-frontal oxygen consumption during simulated (dry) dynamic than static apnea, resulting from a relatively attenuated increase in cerebral blood velocity (Allinger et al. 2025); the simulated nature of the experiment however limits its application to the underwater environment. Despite cerebral vasodilation to increase cerebral blood flow and maintain oxygen delivery, in a circulatory system already polarized towards preserving central perfusion during diving, compensatory mechanisms may be insufficient especially in extreme dives with hypoxia developing during the ascent phase. Since TSI expresses tissue oxygen saturation of hemoglobin—calculated combining arterial and venous contribution—it does not account for the actual oxygen availability at the cellular level. In this context, the mechanisms underlying transient neurological symptoms may involve critical thresholds of oxygen tension within the interstitial space, cytosol, or mitochondria. Individuals retaining sufficient residual peri-cellular or intracellular oxygen during the last meters of ascent might preserve cerebral functions despite marked reductions in TSI, peripheral oxygen saturation, and cerebral blood volume. Such cellular-level, individual variability in oxygen—rather than hemoglobin desaturation *per se*—may therefore be a critical determinant of susceptibility to LMC and blackout, yet unmeasured *in vivo*.

Blood biomarkers, such as S100β and neuron-specific enolase (NSE), increase after diving, indicating subclinical blood-brain barrier disruption and possible glial-neuronal damage. Specifically, increases in S100β suggest transient brain-blood barrier permeability changes rather than permanent neurological damage, though long-term consequences remain uncertain (Linér and Andersson 2009; Janigro et al. 2021). Gren et al. also identified increases in beta-amyloid (Aβ42) and T-tau proteins after static breath-hold, without a concurrent rise in S100β, secondary to hypoxia (n = 16, competitive divers) (Gren et al. 2016).

A neurocognitive study suggests that experienced freedivers (n = 10) maintain cognitive performance and early visual processing during prolonged, dry, static apnea, likely due to neuroadaptive mechanisms despite hypoxia (Steinberg and Doppelmayr 2019). Sensory sensitivity (through critical flicker fusion frequency assessment) was unchanged in 39 experienced divers after a dynamic apnea (surface swimming) (de Asís Fernández et al. 2019a). However, evidence during actual breath-hold dives is still missing.

Overall, research on the neurological implications of breath-hold diving remains limited. Key priorities include clarifying the potential contribution of cardiac arrhythmias to blackouts independent of hypoxia, mechanism of cerebral oxygen regulation, and the cause of transient blood-brain barrier dysfunction. The identified studies offer initial clues, but meaningful progress will require study protocol simultaneously assessing brain, heart, and gas exchange. NIRS application in human breath-hold diving is still experimental; developing portable systems to monitor cerebral TSI could help mitigate blackout risk across all levels of practice. Also, validating blood biomarkers such as S100β and NSE for neurovascular health monitoring could similarly support future point-of-care systems to detect situations at risk and suspend breath-hold activity earlier during competitions. Another priority is to clarify the effects of breath-hold diving on cognitive domains and thereby confirm or refute the occurrence of inert gas narcosis in breath-hold divers. Tracking of symptoms paired with depths can provide evidence for the real incidence of the phenomenon. Finally, future studies should not only mirror gas narcosis theories and studies performed in SCUBA divers, but also include the combined effects of other gases—e.g., hypoxia and hypercapnia—especially at tissue and cellular level.

### Decompression stress and decompression illness risk

Thirty-one manuscripts were assigned to this theme, including 12 original research papers, 10 case reports and one case series, 7 reviews, and one systematic review by Blogg et al. (Blogg et al. 2023).

DCI in breath-hold divers, once considered theoretically impossible, has been increasingly documented, although its pathophysiology remains unknown. Early perspectives largely dismissed the risk of hazardous nitrogen accumulation, identifying shallow dive depths, short dive durations, and limited lung surface as protective factors against DCI (Blogg et al. 2023). However, several reports published over time suggested that even relatively shallow but repetitive dives can dissolve nitrogen in tissues, creating reservoirs that contribute to intravascular bubble formation and decompression-related pathology (Goldman and Solano-Altamirano 2015). Polynesian pearl divers provided some of the earliest anecdotal evidence of a diving-related illness termed *taravana*: dizziness, nausea, confusion, and paralysis affecting divers following prolonged and repeated dives. At the time, these symptoms were attributed to hypoxia rather than decompression stress (Schipke et al. 2006). In the latter half of the 20th century, sporadic cases of neurological and vestibular dysfunction were documented in military divers and spearfishers performing frequent dives to significant depths with minimal surface intervals, but lacked direct imaging or physiological evidence supporting DCI; a theoretical model by Thorsen et al. later predicted which diving profiles carried a risk of DCI (Thorsen et al. 2007).

An important shift occurred in the early 2000s when imaging techniques provided evidence of diving-related ischemic lesions in the brains of breath-hold divers. MRI studies confirmed focal infarctions in regions consistent with embolic events, particularly in “low flow” areas—the brainstem, basal ganglia, and cortical watershed areas– suggesting that nitrogen emboli, rather than hypoxia alone, were responsible for the observed neurological symptoms (Guerreiro et al. 2018; Kohshi et al. 2021a). In the same period, Doppler ultrasound studies demonstrated the presence of venous gas emboli (VGE) after repeated breath-hold dives in 12 male Ama divers (Lemaître et al. 2014), whereas no VGE were present 1 h after completing a fishing competition (11 recreational spearfishermen, average 80 dives/each, to about 20 msw) (Gargne et al. 2012). By the 2010s, computational models adapted from SCUBA decompression algorithms suggested that nitrogen could supersaturate breath-hold divers’ tissues during descent, exceeding DCI thresholds, especially with deep dives, rapid ascent rates, and surface intervals insufficient for adequate off-gassing (Goldman and Solano-Altamirano 2015). Once generated, VGE can manifest locally as decompression sickness (DCS), travel to the lungs to be retained and filtered, or become arterialized if the bubble load overwhelms the pulmonary capillary network’s filtering capacity. Arterial gas embolism (AGE) and DCS present distinct but overlapping pathophysiological mechanisms in breath-hold divers. AGE can occur if venous gas emboli pass through right-to-left shunts, such as intrapulmonary arterio-venous anastomoses (IPAVA) during exercise or hypoxia (Kohshi et al. 2021a), or a patent foramen ovale (PFO), which appears more prevalent in elite breath-hold divers (Kelly et al. 2022), although evidence is still insufficient to confirm PFO as an independent risk factor for AGE (Lovering et al. 2022). Unlike compressed-gas diving, where spinal cord involvement is common, DCS in breath-hold divers primarily manifests in the cerebral circulation with stroke-like symptoms—dizziness, limb weakness, altered mental status, and vestibular, visual and sensory disturbances (Matsuo et al. 2014; Tetzlaff et al. 2017; Accurso et al. 2018; Kohshi et al. 2021a; Mason et al. 2023). Using a mathematical model, Arieli suggested that microbubbles may nucleate autochthonously in small distal arteries (Arieli 2019), providing a possible mechanism for alternative distribution of emboli and confirming distal arterial bubble formation rather than venous-to-arterial shunting alone behind cerebral DCI. Druelle et al. used this hypothesis to partially explain a case of *taravana* and posterior reversible encephalopathy syndrome after repeated breath-hold dives to 40 msw with a scooter, showing vasogenic edema on brain MRI (FLAIR hyperintensities, not mirrored in diffusion-weighed imaging sequences) and not emboli-induced ischemic lesions (no cytotoxic edema)—implicating blood-brain barrier disruption from autochthonously formed bubbles in the pathophysiology (Druelle et al. 2024). A similar mechanism could explain the vasogenic edema on brain MRIs reported in different case reports (Matsuo et al. 2014; Sánchez-Villalobos et al. 2022).

In commercial Ama divers, neurological symptoms following repeated breath-hold dives have been frequently reported, with a higher incidence among Funado (deep divers using weights) divers than among shallow divers (Kohshi et al. 2021b). Diacono et al. report a professional freediver experiencing memory loss and sensory abnormalities, which resolved after hyperbaric oxygen therapy (Diacono and Magri 2022). While AGE remains less common than neurological DCS, some breath-hold divers have presented with sudden loss of consciousness, mimicking SCUBA-related AGE and posing differential diagnosis challenges. Yanagawa et al. describe an elderly Ama diver who developed right hemiparesis after repeated shallow breath-hold dives; the event was diagnosed as a transient ischemic attack of presumed endogenous origin, after no intracardiac bubbles were visualized with point-of-care ultrasound (POCUS) performed on scene (Yanagawa et al. 2018). However, the absence of detectable intracardiac gas bubbles in breath-hold divers presenting with acute neurological symptoms cannot reliably exclude AGE, as no residual bubbles may be detectable once the embolic event has occurred. Tetzlaff et al. summarize a series of 4 stroke-like symptoms after breath-hold diving, improving or resolving after hyperbaric oxygen therapy, with characteristic ischemic lesions on brain imaging, supporting AGE etiology (Tetzlaff et al. 2017); a similar case is reported by Kohshi et al. involving a commercial breath-hold diver (Kohshi et al. 2020). Pulmonary barotrauma after lung overexpansion techniques—such as glossopharyngeal insufflation—has also been correlated with a suspected case of AGE (Linér and Andersson 2010). DCI in breath-hold divers can occasionally present with other systemic symptoms, such as musculoskeletal pain—particularly in the limbs and joints—or cardiovascular discomfort, including palpitations (Kohshi et al. 2021b).

Some divers—particularly long-term professionals—exhibit asymptomatic brain lesions detected via MRI (Kohshi et al. 2020), raising concerns about long-term neurovascular damage from repetitive diving in this subset of individuals. Recently, biomarker-based research offered new insights into subclinical decompression stress and potential prevention strategies. Endothelial dysfunction (Cialoni et al. 2021b, a), oxidative stress and inflammation (Eftedal et al. 2016; Mrakic-Sposta et al. 2019; Sulfiana et al. 2024; Vezzoli et al. 2024), altered immune function (Eftedal et al. 2016; Gašparini et al. 2022), and heat shock protein elevation (Solich-Talanda et al. 2021) observed after dives suggest that even individuals who do not experience acute symptoms may still accumulate physiological damage over time. This cumulative decompression stress may be mitigated by antioxidant supplementation and personalized workload management aiming at reducing long-term cerebral and cardiovascular impact, and monitored through periodical testing if validated for non-experimental contexts.

Future areas of improvement include, first, the validation of DCI as sustaining acute neurological syndromes, testing the competing hypotheses of autochthonous bubble formation in distal cerebral arteries vs. the arterialization of venous bubbles. At present, evidence consists only of case reports including brain imaging, showing either vasogenic edema from locally derived bubble stress (Matsuo et al. 2014; Sánchez-Villalobos et al. 2022; Druelle et al. 2024) or ischemic lesions from AGE (Tetzlaff et al. 2017; Kohshi et al. 2020); a very low level of evidence that is still insufficient to support either mechanism over the other, or to affirm that both can sustain acute neurologic symptoms with different features. Second, the prospective investigation of sensitive biomarkers for long-term monitoring is a promising way to detect decompression stress, especially in professional and military divers. Third, mathematical models dedicated to breath-hold divers that account for lung compression and nitrogen kinetics could help define risk profiles and optimal surface intervals during training, competitions, and professional activity, mitigating acute and chronic DCI risk.

### Skeletal muscle and metabolic adjustments

Seventeen studies—15 original research papers, one case series, and one review by Drviš et al. (Drviš et al. 2025b)—were related to this theme and published in the last two decades.

Breath-hold diving induces significant stress on skeletal muscles, reflected by increased creatine kinase (CK), lactate dehydrogenase (LDH), and cardiac creatine kinase isoenzyme (CK-MBm) (Cialoni et al. 2021c), suggesting muscle microtrauma, anaerobic metabolism, and hypoxic stress due to the strenuous physical activity and pressure-induced peripheral hypoxia associated with deep dives. Significant alterations in circulating amino acid profiles also reflect metabolic demands related to intermittent hypoxia, and a reduction in citrulline levels has been correlated to enhanced nitric oxide (NO) production, contributing to vasodilation and oxygen conservation during dives (Cialoni et al. 2022). Both studies included 12 experienced breath-hold divers during open sea trainings, therefore reflecting real world conditions despite the small sample size.

Patrician and Schagatay show that dietary supplementation with nitrate-rich beetroot juice enhanced arterial oxygen saturation after pool-dives in 14 experienced divers, promoting oxygen conservation and improving mitochondrial efficiency, likely supporting maximal apnea performance (Patrician and Schagatay 2017). Contrarily, Lorei et al. report two cases of blackout and suggest that beetroot powder-induced vasodilation may disrupt the normal cardiovascular diving response, leading to decreased cardiac output and perfusion at depth (Lorei et al. 2024). Ghiani et al. found that overnight fasting after a one-week diet (with 50% carbohydrates, 20% fats) improved the static phase duration on 8 freedivers diving to 30 msw, increased arterial oxygen saturation, and reduced blood lactate levels, compared to similar post-breakfast dives. Potential explanations are the inferior splanchnic pooling of blood in the fasting trial, available to improve the diving response, and less abdominal content causing discomfort and reduced diaphragmatic excursion in the breakfast trial (Ghiani et al. 2016). Breath-hold diving is also linked to increased insulin levels and decreased blood glucose, indicating upregulated glucose uptake not associated with GLUT4 activation, possibly due to intermittent hypoxia and elevated catecholamines after 5 dives to 20 meters (20 experienced divers) (Sponsiello et al. 2018). However, a carbohydrate-free diet and prolonged exercise shifted metabolism towards lipid oxidation, reducing CO₂ production, thus delaying the urge to breathe and exacerbating hypoxia in experienced breath-hold divers performing dry apnea. This brings attention to divers having a lipid-rich diet or carbohydrate depletion after prolonged breath-hold diving (e.g., spearfishing) and the increased risk of losing consciousness (Lindholm and Gennser 2005), despite performing the experiment in dry conditions limits current underwater applications. In a recent review, Drviš et al. suggest a Mediterranean and alkaline diet to meet breath-hold diving’s requirements for acid-base balance and adequate recovery from repeated hypoxic stress, with specific modifications according to the technique (e.g., static vs. dynamic vs. spearfishing) (Drviš et al. 2025b)

Post-apnea lactate accumulation in experienced breath-hold divers suggests significant anaerobic contribution across different exercise protocols: after simulated static and dynamic (dry) breath-holds (Brown et al. 2024; Allinger et al. 2025), dynamic apnea (surface swimming) (Breskovic et al. 2011; Vitali et al. 2022; Drviš et al. 2025a), and pool (Bosco et al. 2020) and open-sea dives (Marongiu et al. 2015). Lactate increases more in disciplines involving large muscle groups and prolonged apneic episodes, such as progressively deeper fin-assisted dives (Marongiu et al. 2015), fins vs. sled-assisted dives (Bosco et al. 2020), and fin vs. no fin dives (Rodriguez-Zamora et al. 2018). Consistently, a shift towards anaerobic metabolism is suggested by the increased oxygen extraction measured in muscles of 19 experienced divers in a simulated (dry) breath-hold—an attempt to compensate for the imbalance between oxygen supply and consumption (Allinger et al. 2025).

Competitive freedivers and spearfishermen (n = 21) exhibit significant muscle oxygenation asymmetry between dominant and non-dominant limbs, which may affect performance and fatigue resistance, emphasizing the need for targeted training (Uljević et al. 2024). In 22 competitive divers, Vinetti et al. found that dynamic apnea has lower energy cost when using a monofin compared to bi-fins, consistent with hydrodynamic theory. Of note, experiments are performed at surface, thus not including effects of pressure, but personal best distances in dynamic apnea with fins correlated more strongly with the meters swum per liter of O_2_ store depleted than with the inverse of energy cost—highlighting the importance of oxygen conservation mechanisms alongside swimming efficiency (Vinetti et al. 2025).

Future research in this field should distinguish the interplay between hypoxia-induced and exercise-induced adaptations on metabolic changes and hormones and dietary interventions to optimize oxygen utilization and fatigue resistance. Availability of participants for such intervention studies could be mitigated by performing crossover trials, which are unfortunately underrepresented in the retrieved literature. The studied biomarkers can constitute—with others already noted in the *Decompression Stress and Decompression Illness Risk* and the *Neurological and Cognitive Effects* sections—a multipanel screening test.

### Training factors

Eight studies—seven original research studies published in the last 5 years and one review by Ostrowski et al. (Ostrowski et al. 2012)—were identified.

Overall, the studies show that training duration and specific methods affect the physiological parameters of breath-hold divers differently. An initial study focusing on medium-term adaptations (22 weeks) combined apnea-specific exercises with physical conditioning on male breath-hold divers (two years of experience), resulting in improved lung capacity, VO_2_max, and underwater swimming economy; apnea training alone did not achieve such result (Fernandez et al. 2019). In a single blind, crossover trial involving young divers, short-term performance-specific training modalities such as inspiratory muscle training (IMT) and high-intensity interval training (HIIT) were shown to improve recovery capacity after dynamic apnea. Also, IMT seemed to enhance recovery by increasing inspiratory muscle strength and reduce perceived exertion, whereas HIIT contributed to improved metabolic efficiency and oxygen utilization (de Asís-Fernández et al. 2020). Increased breath-hold time and changes in hemoglobin oxygen saturation were demonstrated in 10 novice breath-hold divers after two weeks of specific dry, static apnea training (Bourdas and Geladas 2024).

Recent studies constructed and validated sport-specific anaerobic sprint tests on 34 elite and intermediate level breath-hold divers, revealing a substantial predictive value for dynamic apnea performance, suggesting that anaerobic capacity is a critical determinant of competitive success (Drviš et al. 2024). This concept has been further supported by lower lactate levels produced by elite divers (n = 6) after apneic monofin swimming than by intermediate-level divers (n = 9) (Drviš et al. 2025a). Lee et al. identified critical physiological predictors for static apnea duration in 36 male, experienced breath-hold divers, such as lung capacity, extended arterial oxygen saturation (SpO₂) range, and reduced body fat (Lee et al. 2024). Building on previous findings, Drviš et al. found in mixed elite/intermediate breath-hold divers that a combined aerobic–anaerobic training improved dynamic apnea duration, diving-specific anaerobic capacity, and selected swimming performance parameters, with the greatest gains observed during anaerobic training phases—likely due to enhanced anaerobic energy system engagement and improved muscle buffering capacity (Drviš et al. 2025c).

Overall, the available evidence is still insufficient to provide clear directions to improve training. Future research should, therefore, bridge short- and medium-term with long-term physiological adaptations in apnea diving since specialized training also induces structural adaptations, such as increased lung volume, capillary density, and hematological changes, leading to improved oxygen utilization efficiency (Ostrowski et al. 2012)—as detailed in the following section. Also, more attention should be given to less-experienced divers (included in only one study), to identify best training strategies and mitigate risk of injury in those starting this sport.

### Long-term physiological adaptations

Nineteen papers were included—18 original research papers and a seminal review by Elia et al. (Elia et al. 2021). Long-term exposure to breath-hold diving induces complex physiological changes, conferring resilience against environmental stressors but potentially translating into harmful adaptations.

The cardiovascular system undergoes an enhanced diving response that improves oxygen sparing by reducing cardiac output and prioritizing blood flow to vital organs such as the brain and heart. Scherhag et al. did not find significant pathological patterns among eight competitive breath-hold divers, but only potential early signs of right ventricular strain on ECG, 2-D and doppler echocardiography (Scherhag et al. 2005). Consistently, higher left ventricle and atrium volumes (within normal ranges) and right ventricle volume slightly above normal values were found in 16 well-trained breath-hold divers compared to matched, non-sportive controls, as a probable adaptation to central blood pooling (Zelenkova and Chomahidze 2016). Despite no observed long-term structural changes, acute cardiac strain, transient elevations in cardiac stress biomarkers, and left ventricular dilation during maximal apnea attempts suggest an accumulation of silent damage (Elia et al. 2021).

During exercise with face immerged, a faster onset of bradycardia and more pronounced peripheral vasoconstriction were measured in 14 well-trained divers compared to 14 non-divers (Tocco et al. 2012). Consistently, peripheral resistance increased more in 15 well-trained breath-hold divers than in controls during a 30-s breath-hold with immersed face (Peng et al. 2021). In a large cohort of lifelong Ama fisherwomen, Sugawara et al. showed significantly lower stiffness in proximal arteries, lower carotid impedance, and superior aortic reservoir function than age-matched non-divers; however, the consequences on cerebrovascular health were not investigated (Sugawara et al. 2018). Park et al. demonstrated in 10 Korean Haenyeo divers enhanced cold tolerance compared to non-diving women of the same age, as a potential consequence of peripheral vasoconstriction and a higher percentage of subcutaneous fat against cold temperatures (Park et al. 2018). Recent findings by Aguilar-Gómez confirm higher diastolic blood pressure values in 30 Haenyeo divers and in 30 Jeju islanders vs. mainland residents, accompanied by a genetic variant to significantly reduce diastolic blood pressure, potentially representing a positive selection to mitigate hypertensive risks in female divers during pregnancy (Aguilar-Gómez et al. 2025). Long-term breath-hold diving has also been associated with bradycardia at rest (Zelenkova and Chomahidze 2016; Peng et al. 2021) and during diving (Aguilar-Gómez et al. 2025). An increased incidence of cardiac arrhythmias—including supraventricular arrhythmias and atrioventricular blocks—was demonstrated and attributed to extreme hypoxia and hypercapnia-induced autonomic imbalances, only on 8 of the 16 included well-trained divers (Zelenkova and Chomahidze 2016). Splenic contraction is also enhanced in this population, increasing hemoglobin concentration and boosting oxygen-carrying capacity during dives, as found by Holmström et al. on 12 elite biathletes vs. controls during simulated apnea (Holmström et al. 2021).

The respiratory system adapts to improve capacity and efficiency, enhancing oxygen storage and utilization during prolonged apnea. Walterspacher et al. demonstrated significantly greater lung volumes—including TLC and VC—in 12 elite breath-hold divers than in non-divers, an effect mainly attributed to the routine use of GI. In a three-year follow-up of four of these divers, pulmonary compliance and ventilatory flows appeared unchanged; the authors interpreted it as a benign and effective adaptation, but the limited sample size precludes firm conclusions (Walterspacher et al. 2011). Ferretti et al. confirmed TLC values up to 22% higher than predicted for their body size in 8 elite breath-hold divers (Ferretti et al. 2012). Consistently, Diniz et al. found increased FVC and FEV1 in 11 fishermen compared to 10 non-divers, indicating enhanced lung volumes without airway obstruction (Diniz et al. 2014). Solich-Talanda et al. confirmed more recently an increase in VC, FEV1, and maximal expiratory flow on 17 well-trained divers (Solich-Talanda et al. 2019‬‬‬‬)‬.‬‬‬‬‬‬ ‬‬‬‬‬‬‬‬‬‬‬‬Findings on bone mineral density (BMD) and content are conflicting. Hwang et al. found that BMD in the proximal femurs of 61 divers was higher than in matched controls, but declined more rapidly with age. This pattern suggests that young divers may benefit from greater skeletal loading during deeper, more effortful dives, while, in older divers, BMD deteriorates more markedly—potentially due to less strenuous activity and prolonged exposure to buoyancy, resembling chronic unloading (Hwang et al. 2006). Seo et al. suggested no difference between breath-hold and non-diver post-menopausal populations, but on two smaller and unmatched cohorts (Seo et al. 2018). Variations might be due to differences in measurement sites and populations (Elia et al. 2021). Additionally, an increased prevalence of chronic kidney disease was observed in a large cohort of Haenyeo divers (Oh et al. 2017).

The term “maladaptations” has recently been connected with breath-hold diving to highlight potential long-term damage rather than physiological adaptation. A seminal work by Elia et al. identifies several other fields needing investigation (Elia et al. 2021), such as neurocognitive impairment with evidence of small cerebral ischemic lesions, neuronal damage biomarkers, and cognitive deficits. Billaut et al. found that elite breath-hold divers are at risk for mild but persistent short-term memory impairments, suggesting intermittent hypoxia-induced neuronal damage as the cause potentially affecting executive functions related to inhibitory control and working memory (Billaut et al. 2018). Whether this damage is caused by systemic hypoxemia or by AGE causing local hypoxia remains to be determined. Early research on a small sample showed that elite breath-hold divers maintain cerebrovascular reactivity to hypercapnia despite repeated apnea exposure (Ivancev et al. 2007). However, recent evidence suggests that while static cerebral autoregulation is preserved, dynamic cerebral autoregulation is impaired. Seventeen elite breath-hold divers exhibited a slower rate of regulation and prolonged cerebral vasodilation when exposed to hypotension through a sit-to-stand protocol, increasing the risk of cerebral hypoperfusion and ischemia during transient hypotensive events and making this field still poorly explored (Moir et al. 2019).

Overall, several gaps persist. Studies including lifelong divers are the most effective models, suggesting positive adaptations especially in the cardiovascular domain, but are affected by genetic predispositions, gained through centuries of selection pressure, and are not completely applicable to the general breath-hold diver population. Other identified studies focus on divers with years of practice, but unfortunately include a small number of participants, thus limiting the conclusions regarding potential cardiac strain and predisposition to arrhythmias or cognitive impairment. Experienced breath-hold divers proved to have higher pulmonary function values than the average population, but only one study attempted a short-term (3 years) follow-up therefore was not able to achieve a substantial conclusion on harmful changes over time. It is clear that, to shed light on long-term adaptations, the priority is improving the quality of pertinent findings by establishing a robust transdisciplinary and multicountry follow-up.

### Telemonitoring and technological advancements

Five manuscripts—four original studies, and a systematic review by Vinetti et al. (Vinetti et al. 2020)—were connected to this theme, published in the last 4 years (2021–24), suggesting this is still unexplored and potentially the most innovative across the eight identified themes.

The advent of underwater pulse oximetry paved the way for continuous monitoring of SpO₂ and heart rate in breath-hold divers. Mulder et al. validated a submersible pulse oximeter in static and dynamic apnea conditions, demonstrating its ability to track desaturation events with a response time superior to traditional, non-immersible transmission pulse oximeters (Mulder et al. 2021, 2023b).With a non-invasive technique, their findings confirmed that SpO_2_ levels can drop below 50% during ascent in deep freedives, a phenomenon previously inferred but not empirically measured in real-time until arterial blood gas analysis sampling already described in the “*Respiratory System and Gas Exchange*” section. Despite these advancements, motion artifacts and data loss due to sensor displacement or signal disruption continue to limit pulse-oximeter reliability. Wu et al. introduced a self-calibrated pulse oximetry algorithm designed to enhance the accuracy of SpO_2_ measurements by compensating for variations in photon pathlength and tissue absorption and improved measurement fidelity at low SpO₂ levels (since conventional pulse oximeters are typically calibrated down to 80% saturation) (Wu et al. 2023).

The use of wearable sensors in freediving has expanded beyond that, incorporating accelerometers, piezoelectric sensors, and stretch sensors to provide a broader physiological profile. These devices have been employed to track HRV, ventilation patterns, and body movement to improve safety and performance (Vinetti et al. 2020). Specifically, validating such technologies could offer the potential for early detection of distress in breath-hold divers and a way to assess individual susceptibility to hypoxia and optimize training regimens. However, a major challenge is to adapt devices developed for terrestrial or clinical settings to the extreme environmental conditions of freediving, such as rapid changes in pressure, temperature, and blood flow redistribution, or practical challenges related to sensor adherence, waterproofing, and data integrity at depth.

More recently, hyperventilation detection devices have been tested to prevent this well-documented risk factor for hypoxic blackout. By developing a novel force sensor technology, Pernett et al. demonstrated that changes in respiratory amplitude and frequency could be used to estimate pre-dive CO_2_ levels with moderate accuracy, offering a potential solution for identifying divers who may be at heightened risk of blackout due to excessive hyperventilation (Pernett et al. 2024a). However, the system explained only 34% of the variability in end-tidal CO₂ and was tested exclusively in dry, static apnea conditions; thus, its efficacy and reliability in dynamic underwater environments remain to be investigated.

The technological advancements outlined in these studies collectively represent a shift toward more precise, real-time monitoring solutions for freediving. Yet most systems remain in an early developmental phase, with limited real-world testing and little evidence that the recorded signals can reliably predict adverse events. Future work should prioritize measurement accuracy, usability, and robust real-time data transmission from depth to the surface for proper telemonitoring. Only once these steps are achieved it will be possible to integrate telemonitoring tools into pragmatic safety protocols for all-level breath-hold divers.

### Beyond the themes: an integrated view of the findings

Taken together, dividing the literature into themes clarifies the advances over the past two decades and the persisting gaps. However, clinically relevant outcomes of breath-hold diving cannot be understood by examining cardiovascular, pulmonary, neurological and other responses in isolation. Real-world dives combine hydrostatic pressure, hypoxia–hyperoxia cycles, cold exposure, apneic exercise, and repetitive diving, often with techniques such as GI. These stressors act simultaneously on multiple systems, triggering cascades that propagate from one compartment to another and ultimately present as acute or chronic, clinical or subclinical syndromes.

One illustrative example is loss of consciousness (syncope). According to the literature, the leading hypothesis attributes syncope to acute cerebral hypoxia from pulmonary or cardiovascular involvement. Over repeated dives, central blood shifting with rise in cardiac-vascular pressures and cardiac volumes alterations (e.g., reduced cardiac output) can lead to pulmonary edema, alveolar compression/re-expansion stress to atelectasis, and extreme swings in airway pressures to barotrauma (from GI or negative spikes during involuntary diaphragmatic contractions); all these mechanisms potentially impair alveolar—capillary integrity and gas exchange, resulting in hypoxemia. Another point of view includes diving-induced altered cerebral blood flow regulation and autonomic control, suddenly reducing brain perfusion. Finally, complex interactions between immersion, cold shock, chemoreflex activation, and strong vagal drive may generate autonomic conflict and malignant arrhythmias, suppressing blood flow. From a pulmonary perspective, worrisome manifestations such as hemoptysis may arise from extreme fluctuations in airway pressure and increased cardiovascular pressures, particularly in the pulmonary vasculature.

Other syndromes include *taravana* and stroke-like presentations. The prevailing hypothesis implicates nitrogen uptake during repetitive deep dives without sufficient surface intervals. However, the mechanism of injury remains unclear, with two main possibilities: peripheral venous gas emboli that bypass the pulmonary filter because of underwater cardiovascular adaptations, or blood-brain barrier damage from locally (cerebral) generated bubbles or microbubbles. Cognitive impairment remains poorly studied, and the incidence of nitrogen narcosis is debated.

The core message is that research in the coming decades should be driven by clinically meaningful questions and adopt multi-system underwater investigations to achieve concrete results. With technological developments, real-time monitoring during actual dives will be feasible and should be implemented to strengthen external validity and refine safety recommendations for breath-hold divers at all levels.

## Conclusion

Breath-hold diving represents a unique model to study human adaptations to extreme environments. Despite an important body of literature generated in the last 20 years, critical gaps remain in each of the identified areas, particularly regarding cardiovascular-respiratory adaptations individual variability, decompression stress, neurovascular subclinical damage, and long-term potential damage. By focusing future investigations in these areas, integrating cutting-edge technologies to both provide better physiological insights and potentiate telemonitoring, will not only improve diver safety but also expand our knowledge of human performance limits underwater.

## Supplementary Information

Below is the link to the electronic supplementary material.

Supplementary file1 (DOCX 12 kb)

Supplementary file2 (DOCX 18 kb)

Supplementary file3 (DOCX 41 kb)

## Data Availability

Data availability is not applicable to this article as no new data were created or analysed in this study, just reported from the included manuscripts.

## Abbreviations

ABG: Arterial blood gas
AGE: Arterial gas embolism
APC: Adenosine plasma concentration
BMD: Bone mineral density
CK: Creatine kinase
DCI: Decompression illness
DCS: Decompression sickness
FEV1: Forced expired volume in one second
FVC: Forced vital capacity
GI: Glossopharyngeal insufflation
HIIT: High-intensity interval training
HRV: Heart rate variability
IMT: Inspiratory muscle training
IPAVA: Intrapulmonary arterio-venous anastomoses
LDH: Lactate dehydrogenase
LMC: Loss of motor control
MAP: Mean arterial pressure
MRI: Magnetic resonance imaging
NO: Nitric oxide
NSE: Neuron-specific enolase
PFO: Patent foramen ovale
SpO₂: Arterial oxygen saturation
TLC: Total lung capacity
TSI: Tissue saturation index
VC: Vital capacity
VGE: Venous gas emboli
