# Supplementary material for: Advances in breath-hold diving research: a state-of-the-art review
Source: Eur J Appl Physiol. 2025 Dec 19;126(3):1223–43. doi: 10.1007/s00421-025-06093-6 (PMC13013280; doi:10.1007/s00421-025-06093-6)
Supplement: Supplementary file 1 — Supplementary file1 (DOCX 12 kb) [file 421_2025_6093_MOESM1_ESM.docx]

**Supplementary File 1**: Strings used for database scanning

PUBMED

("Breath-hold diving" OR "Freediving" OR "Apneic diving") NOT ("scuba diving" OR "Technical Diving" OR "Rebreathers") AND (("2004/01/01"[Date - Publication] : "3000"[Date - Publication])) AND (english[Filter]) NOT (animal[Filter])

SCOPUS

( TITLE-ABS-KEY ( "Breath-hold diving" OR "Freediving" OR "Apneic diving" ) ) AND NOT ( TITLE-ABS-KEY ( "Scuba Diving" OR "Technical Diving" OR "Rebreathers" ) ) AND NOT ( TITLE-ABS-KEY ( "dolphin*" OR "whale*" OR "seal*" OR "sea lion*" OR "otter*" OR "porpoise*" OR "turtle*" ) ) AND ( PUBYEAR > 2003 ) AND ( LIMIT-TO ( LANGUAGE , "English" ) ) AND ( LIMIT-TO ( DOCTYPE , "ar" ) OR LIMIT-TO ( DOCTYPE , "re" ) OR LIMIT-TO ( DOCTYPE , "le" ) OR LIMIT-TO ( DOCTYPE , "ch" ) )
