# Supplementary material for: Advances in breath-hold diving research: a state-of-the-art review
Source: Eur J Appl Physiol. 2025 Dec 19;126(3):1223–43. doi: 10.1007/s00421-025-06093-6 (PMC13013280; doi:10.1007/s00421-025-06093-6)
Supplement: Supplementary file 2 — Supplementary file2 (DOCX 18 kb) [file 421_2025_6093_MOESM2_ESM.docx]

**Supplementary Table 2:** Thematic Analysis Guide, with definitions (in italic).

| Themes | Subthemes | |
| --- | --- | --- |
| Cardiovascular and Hemodynamic Adaptations  *Analyzes the physiological adjustments of the cardiovascular system (heart and blood vessels) in response to breath-hold diving.* | Diving Response  and Autonomic Regulation  *The autonomic responses activated by apnea and/or immersion, aiming at optimizing oxygen conservation and prolonging underwater time.* | |
|  | Splenic Contraction and Hematological Adjustments  *The mobilization of erythrocytes from the spleen to increase oxygen transport capacity during apnea, prolonging dive duration.* | |
|  | Blood Pressure, Perfusion and Vascular Responses  *Acute hemodynamic effects of breath-hold diving, including arterial blood pressure variations, vascular compliance changes, direct and indirect effects on cardiac output.* | |
| Respiratory System and Gas Exchange  *Explores pulmonary adaptations, gas exchange dynamics, and respiratory mechanics during breath-hold diving.* |  |  |
|  | Lung Volume Dynamics and Adaptations  *Adjustments in static and dynamic lung volumes, compliance, in response to environmental pressure variations and preparation techniques in breath-hold diving.* | |
|  | Pulmonary Barotrauma (“Lung Squeeze”) and Edema  *Pathophysiological consequences of pressure changes during breath-hold diving affecting the lungs, including alveolar compression/expansion stress, atelectasis, lung edema, and hemoptysis.* | |
|  | Oxygen Storage and Utilization  *The distribution and utilization of oxygen reserves across pulmonary and blood compartments to optimize hypoxia tolerance.* | |
|  | Hypercapnia Tolerance and CO_2_ Buffering  *The physiological mechanisms underlying an increased tolerance to elevated CO₂.* | |
| Neurological and Cognitive Effects  *Investigates the impact of breath-hold diving on neurological and cognitive functions.* |  |  |
|  | Hypoxia and Ascent Syncope (“Blackout”)  *Loss of consciousness during breath-hold diving, particularly during ascent, and its potential causes (hypoxia or other emerging theories).* | |
|  | Cerebral Autoregulation and Neuroprotection  *The capacity of the brain to regulate blood flow despite hemodynamic variations induced by breath-hold diving, to preserve function and oxygenation.* | |
|  | Cognitive impairment  *Neurophysiological impairment at depth, leading to, e.g., cognitive dysfunction and altered motor coordination.* | |
| Decompression Stress and Decompression Illness Risks  *Examines the pathological consequences of inert gas accumulation, decompression illness, and stress generated by decompression and repetitive diving in breath-hold divers.* |  |  |
|  | Inert Gas Accumulation and Microbubble Formation  *The absorption in tissues of nitrogen at pressure and its subsequent release as microbubbles during ascent, potentially leading to decompression illness.* | |
|  | Neurological Manifestations of Decompression Sickness  *The neurological symptoms associated with decompression stress, including overt (e.g., loss of motor control also called “Taravana”, transient sensitive or motor impairment) or less obvious manifestations (e.g., vestibular disturbances, musculoskeletal pain).* | |
|  | Biomarkers of Decompression Stress  *Biomarkers of stress caused by single or repeated breath-hold diving, leading to subtle and subclinical acute modifications.* | |
| Skeletal Muscle and Metabolic Adjustments  *Analyzes the metabolic and muscular adaptations facilitating prolonged breath-hold diving and anaerobic endurance.* |  |  |
|  | Muscle Metabolism  *Metabolic adaptations of muscles (e.g., mitochondria) to improve efficiency during breath-hold diving.* | |
|  | Dietary Intake Influence  *Modifications of performance or metabolic indicators with specific supplementations or dietary regimens.* | |
|  | Lactate Dynamics  *The role and adaptations of anaerobic metabolism in breath-hold tolerance, lactate accumulation, and buffering capacity.* | |
| Training Factors  *Explores training-related variables influencing breath-hold diving performance and physiological resilience.* | Short- and Medium-Term Training  *The neuromuscular plasticity and physiological modifications resulting from apnea training, enhancing performance, metabolic efficiency, and/or autonomic control.* | |
| Long-Term Physiological Adaptations  *Targets chronic physiological and/or pathological changes induced by repetitive exposure to gas and pressure variations while practicing breath-hold diving for prolonged times.* |  |  |
|  | Cardiovascular and Pulmonary Remodeling  *Long-term cardiac, vascular, and lung adaptations, including e.g., myocardial remodeling, autonomic modulation, static and dynamic pulmonary volumes changes.* | |
|  | Bone Density and Renal Function  *Impact of repeated breath-hold diving on bone remodeling, mineral metabolism, and renal filtration efficiency.* | |
|  | Maladaptations  *Potential long-term negative effects on breath-hold divers, from cardiovascular disease to neurocognitive impairment.* | |
| Telemonitoring and Technological Advancements  *Investigates the role of emerging technologies in tracking performance, monitoring safety, and investigating physiological variations.* |  |  |
|  | *Potential applications of advanced technology to better understand diving physiology, assist athletes’ training, and improve diving safety during trainings and competitions.* | |
