# Supplementary material for: Advances in breath-hold diving research: a state-of-the-art review
Source: Eur J Appl Physiol. 2025 Dec 19;126(3):1223–43. doi: 10.1007/s00421-025-06093-6 (PMC13013280; doi:10.1007/s00421-025-06093-6)
Supplement: Supplementary file 3 — Supplementary file3 (DOCX 41 kb) [file 421_2025_6093_MOESM3_ESM.docx]

**Supplementary Material 3: Study Identification Diagram**

Records identified from Scopus and PubMed

(n = 381)

Duplicate records removed before screening

(n = 194)

**Identification**

**Screening**

Records excluded – not complying with inclusion criteria

(n = 26)

Records screened

(n = 187)

Studies included after screening

(n = 161)

Studies added after a final search before submission

(n = 5)

**Included**

**Total studies included in the review (n = 166)**

Studies included per topic (According to the Guide in Supplementary 2):

- **Cardiovascular and Hemodynamic Adaptations** (n = 46)

- **Respiratory System and Gas Exchange** (n = 40)

- **Neurological and Cognitive Effects** (n = 14)

- **Decompression Stress and Decompression Illness Risk** (n = 31)

- **Skeletal Muscle and Metabolic Adjustments** (n = 17)

- **Training Factors** (n = 8)

- **Long-Term Physiological Adaptations** (n = 19)

- **Telemonitoring and Technological Advancements** (n = 5)

* Studies could be coded to ≥1 topic; totals may exceed 166 *

**Topic Assignment**
